# Integrative single-cell and spatial transcriptomic approaches to decipher the tumor microenvironment and therapeutic resistance in pancreatic cancer

**DOI:** 10.1080/15384047.2026.2699508

**Published:** 2026-07-07

**Authors:** Mengting Luo, Feng Shen, Wanli Xu, Christopher Corpe, Jin Wang

**Affiliations:** a Central Laboratory, Zhongshan Hospital (Xiamen), Fudan University, Xiamen, People's Republic of China; b Department of Medical Oncology, Zhongshan Hospital (Xiamen), Fudan University, Xiamen, People's Republic of China; c Department of Nutritional Sciences, King's College London, London, United Kingdom

**Keywords:** Pancreatic cancer, scRNA-seq, spatial transcriptomics, resistance, TME

## Abstract

Pancreatic ductal adenocarcinoma (PDAC) is among the most aggressive human malignancies and has an extremely poor prognosis. Its progression is largely driven by a highly complex and immunosuppressive tumor microenvironment (TME), highlighting the urgent need for a deeper understanding of its molecular mechanisms. Recent advances in single-cell RNA sequencing (scRNA-seq) and spatial transcriptomics (ST) have provided unprecedented opportunities to dissect cellular heterogeneity, spatial organization, and gene expression dynamics within the TME. In this review, we summarize the major scRNA-seq and ST technologies and their unique strengths in cancer research and highlight their integrated applications in revealing PDAC heterogeneity, stromal–immune interactions, and mechanisms of therapeutic resistance. We further discuss how these approaches can inform biomarker discovery and guide the development of novel therapeutic strategies. Together, these findings suggest that integrated single-cell and spatial transcriptomics offers transformative potential to advance precision oncology and improve outcomes for patients with pancreatic cancer.

## 1. Introduction

Pancreatic ductal adenocarcinoma (PDAC) is among the most lethal cancers worldwide, with a 5-year relative survival rate of only 13.3%.^1^ ^,^ ^2^ PDAC accounts for nearly 90% of all pancreatic cancers and is characterized by rapid progression and high mortality. Surgical resection remains the primary curative option and is usually combined with chemotherapy and/or radiotherapy, whereas immunotherapy has shown only limited benefit.^3^ Owing to the lack of early symptoms and reliable detection strategies, most patients are diagnosed at advanced or metastatic stages, with a median survival of approximately six months.^4^ Over the past decade, advances in sequencing technologies have revealed remarkable transcriptional diversity within PDAC, and transcriptomic approaches provide insights into cellular states, regulatory pathways, and functional heterogeneity within complex tissues. Single-cell RNA sequencing (scRNA-seq) has emerged as a powerful approach for the unbiased characterization of tumor and stromal cell populations, uncovering extensive intratumor heterogeneity and providing valuable insights for prognostic assessment and clinical stratification.^5^ The application of scRNA-seq to patient-derived tumors has revealed distinct malignant subpopulations and highlights the dynamic cross-talk among tumor, stromal, and immune cells within the tumor microenvironment (TME).^6^ From the earliest stages of PDAC, oncogenic events promote the recruitment of stromal and immune components, establishing an immunosuppressive niche that promotes tumor progression.^7^ However, increasing evidence indicates that the TME plays key roles in shaping PDAC initiation, progression, and therapeutic resistance. Unlike other solid tumors, PDAC is characterized by an exceptionally dense desmoplastic stroma composed of cancer-associated fibroblasts (CAFs), extracellular matrix components, and diverse immune populations.^8^ This complex microenvironment not only limits effective drug delivery but also actively regulates tumor cell plasticity, immune evasion, and metastatic dissemination. Consequently, comprehensive analysis of the TME can provide critical insights into the cellular composition, functional states, and intercellular communication networks involved in PDAC progression and therapeutic response.

The poor response of PDAC to immunotherapy is largely attributed to its low mutational burden and predominance of immunosuppressive myeloid cells, which restrict CD8⁺ T-cell infiltration and activation.^9^ High-throughput scRNA-seq enables the profiling of thousands of cells per sample, capturing tumor heterogeneity and the cellular complexity of the TME.^10^ ^,^ ^11^ However, conventional scRNA-seq requires tissue dissociation, which inevitably disrupts spatial information and hinders the study of cell‒cell interactions and tissue architecture. The spatial organization of tumor, stromal, and immune cells within the TME plays a critical role in shaping intercellular communication, immune infiltration, and therapeutic response in PDAC. Cells occupying distinct spatial niches may exhibit different transcriptional programs and functional states despite sharing similar molecular profiles. Therefore, preserving the spatial context is essential for accurately understanding the functional architecture of the PDAC microenvironment. To address this limitation, spatially resolved transcriptomic methods, including paired-cell sequencing strategies, advanced computational algorithms, and direct spatial transcriptomics (ST) platforms have been developed and were shown in Table 1, which preserves spatial context while enabling gene expression profiling, thereby complementing scRNA-seq and offering unprecedented opportunities to study PDAC biology at single-cell resolution in situ.

**Table 1.** Comparison of representative single-cell and spatial transcriptomic technologies.

Technology	Assay type	Resolution	Spatial information	Key advantages	Limitations
Bulk RNA-seq	Population transcriptomics	Tissue-level	No	Provides global gene expression profiles and enables molecular subtype classification	Cannot resolve cellular heterogeneity
scRNA-seq	Single-cell transcriptomics	Single-cell	No	Resolves cellular heterogeneity and identifies rare cell populations	Tissue dissociation leads to loss of spatial context
snRNA-seq	Single-nucleus transcriptomics	Single nucleus	No	Compatible with frozen tissues and difficult-to-dissociate samples	Lower transcript capture efficiency compared with scRNA-seq
10x Visium spatial transcriptomics	NGS-based spatial transcriptomics	Multicellular spot (~55 µm)	Yes	Preserves tissue architecture and enables genome-wide spatial gene expression profiling	Resolution lower than single-cell level
Slide-seq/Slide-seqV2	Bead-based spatial transcriptomics	Near single-cell (~10 µm)	Yes	Higher spatial resolution compared with early ST platforms	Technically complex and lower transcript capture
MERFISH/seqFISH	Imaging-based spatial transcriptomics	Single-cell or subcellular	Yes	Very high spatial resolution and direct visualization in situ	Limited gene panel compared with RNA-seq

## 2. Single-cell sequencing and spatial transcriptome analyses provide new insights into cancer research

Traditional bulk RNA transcriptome sequencing (bulk RNA-seq) captures only average gene expression across populations, thereby masking critical heterogeneity in tumors such as PDAC. The advent of scRNA-seq, first reported in 2009, overcame this limitation by profiling transcription at single-cell resolution.^12^ ^,^ ^13^ Early methods included single-cell tagged reverse transcription sequencing (STRT-seq),^14^ cell expression by linear amplification and sequencing (CEL-seq),^15^ and a switching mechanism at the 5’ end of the RNA transcript (Smart-seq),^16^ with Smart-seq2 further improving sensitivity and cost-effectiveness.^17^ High-throughput platforms such as massively parallel scRNA-seq (MARS-seq),^18^ single-cell RNA barcoding and sequencing (SCRB-seq),^19^ droplet sequencing (Drop-seq),^20^ indexing droplets (InDrop),^21^ and Seq-Well^22^ have enabled parallel profiling of thousands of cells. The 10x Genomics platform^23^ has since become the most widely used system for large-scale scRNA-seq analysis. Till now, scRNA-seq has been widely applied to dissect intratumoral heterogeneity and identify rare malignant and immune cell populations and has revealed distinct malignant cell states and diverse stromal and immune subpopulations in PDAC, providing insights into tumor progression, immune evasion, and therapeutic resistance.^24^ Despite these advances, a major limitation remains: tissue dissociation disrupts the spatial context, restricting insights into cell‒cell interactions and tissue architecture.^25^ ^,^ ^26^

Spatial transcriptomic methods address this limitation by preserving positional information. They are generally divided into imaging-based approaches and next-generation sequencing (NGS)-based strategies.^27-29^ Imaging-based techniques such as multiplexed FISH,^30^ multiplexed error-robust fluorescence (MERFISH),^31^ ^,^ ^32^ seqFISH,^33^ osmFISH,^34^ and HT-smFISH^35^ achieve single-molecule resolution but are often limited by low throughput and probe dependence, as they rely on predesigned, gene-specific probes and thus are inherently restricted to targeted panels rather than unbiased transcriptome-wide profiling. In situ sequencing (ISS) methods, including spatially resolved transcript amplicon readout mapping (STARmap)^36^ ^,^ ^37^ and BaristaSeq,^38^ directly sequence transcripts in situ, enabling high-resolution gene mapping. However, these methods require gene-by-gene analysis and rely primarily on targeted probes to measure a predetermined set of genes, making them time-consuming and low throughput.^39^ In contrast, NGS-based approaches offer high-throughput, cost-efficient solutions. First introduced by Stahl et al. in 2016,^40^ ^,^ ^41^ these methods use spatial barcoding on oligonucleotide arrays, allowing unbiased transcriptome-wide mapping. Representative techniques include Geo-seq,^42^ Slide-seq,^43^ DBiT-seq,^44^ Seq-scope,^45^ sci-Space,^46^ HDST,^47^ and Stereo-seq.^48^ These methods provide higher throughput but often with lower resolution than imaging-based techniques do. Spatial transcriptomic approaches have been increasingly used to map tumor architecture and characterize spatially organized cellular niches and have enabled the identification of multicellular communities and spatial interactions between tumor, stromal, and immune cells, offering critical insights into the mechanisms underlying tumor progression and treatment response in PDAC and other solid tumors.

**Figure 1.**
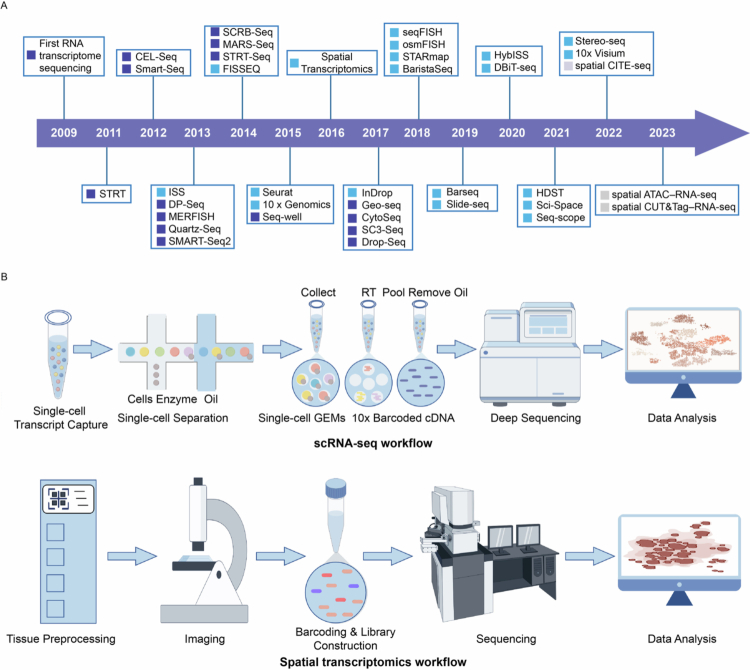
Evolution and workflow of single-cell RNA sequencing and spatial transcriptome. A) The evolution of single-cell RNA sequencing and spatial transcriptomics. The blue boxes indicate scRNA-seq technologies, the purple boxes represent spatial transcriptomic approaches, and the gray boxes denote emerging or multimodal transcriptomic methods. B) Workflow of 10 × scRNA-seq and spatial transcriptomics (by Figdraw). scRNA-seq workflow: First, single cells are isolated and encapsulated into nanoliter droplets together with barcoded beads to form gel bead-in-emulsions. Within each droplet, cells are lysed, and mRNAs are captured and reverse-transcribed into barcoded cDNA, followed by library preparation, high-throughput sequencing, and downstream data analysis (top). Spatial transcriptomics workflow: First, tissue sections are prepared and imaged to preserve spatial context. Spatially resolved barcoding is then performed through either in situ hybridization or spatially indexed capture arrays, followed by library construction, sequencing, and computational reconstruction of spatial gene expression patterns (bottom).

The integration of scRNA-seq and ST has provided a powerful framework to dissect tumor heterogeneity and spatial organization simultaneously. For example, combined approaches have been applied in PDAC and esophageal squamous cell carcinoma, enabling multimodal intersection analysis (MIA) to map distinct subpopulations to defined tissue regions,^49^ ^,^ ^50^ which linked transcriptional cell states to their spatial context, thereby revealing how specific cellular niches contribute to tumor progression and immune regulation. Similar strategies have revealed tumor–stroma interactions in prostate cancer,^51^ neuronal subtypes in the brain,^32^ and developmental processes in cardiac morphogenesis.^42^ Integration of laser capture microdissection with scRNA-seq has further advanced spatial resolution in PDAC tissues, enabling more precise mapping of gene expression within defined anatomical regions.^52^ Thus, these integrative approaches provide critical insights into the spatial architecture of tumors and the functional interactions between malignant and nonmalignant cells, thereby facilitating the identification of novel therapeutic targets and improving our understanding of cancer biology (Figure 1).

## 3. Applications of scRNA-seq and spatial transcriptomics in pancreatic cancer

### 3.1. Tumor heterogeneity in PDAC

PDAC is characterized by remarkable intratumoral heterogeneity, which contributes to its aggressiveness and treatment resistance.^53^ Transcriptomic classification of PDAC has greatly advanced our understanding of disease subtypes and therapeutic responses (Table 2). Early bulk RNA-seq studies established foundational PDAC subtypes. Collisson et al.^54^ identified classical, quasimesenchymal, and exocrine-like subtypes, revealing subtype-specific drug responses to gemcitabine.^54^ Moffitt et al.^55^ used nonnegative matrix factorization (NMF) to define classical, basal-like, normal, and activated types.^55^ Bailey et al.^56^ integrated genomic data from 456 PDACs to identify four groups: squamous, progenitor, immunogenic, and aberrantly differentiated endocrine exocrine (ADEX).^56^ Subsequent studies by Puleo et al.^57^ and Chan-Seng-Yue et al.^58^ further refined the classifications, incorporating stromal and immune compartments.^57^ ^,^ ^58^ All these molecular subtypes are closely associated with distinct clinical outcomes and therapeutic responses. The classical and pancreatic progenitor subtypes are generally linked to a more favorable prognosis and improved response to standard chemotherapy regimens, including FOLFIRINOX or 5-fluorouracil-based therapies; the basal-like or squamous subtypes are associated with poor survival outcomes and increased resistance to conventional treatments; and the immunogenic subtypes exhibit high immune cell infiltration and may show enhanced responsiveness to immune checkpoint blockade therapies (Table 2). Therefore, although the bulk sequencing has limitations in identifying which specific cell types exhibit abnormal gene expression, making pinpointing potential therapeutic targets challenging, scRNA-seq overcomes this limitation by resolving tumor heterogeneity at single-cell resolution.^59^ Bernard V et al. (2018) profiled 5,400 single cells from intraductal papillary mucinous neoplasm (IPMN) and PDAC, identifying epithelial and stromal clusters that captured early malignant transitions overlooked by bulk data.^60^ Peng et al.^24^ delineated two ductal cell types and reported that type 2 ductal cells expressed poor prognosis markers (KRT19 and CEACAMs) and were further subdivided into seven subclusters with roles in proliferation, immune activation, and migration.^24^ ^,^ ^61^ ^,^ ^62^ Additional studies revealed that invasive basal-like clusters are more widespread across PDAC tumors than previously recognized and are consistently detected across all the examined tumors and coexist with classical epithelial programs within individual tumors, although they typically constitute a relatively small fraction of malignant cells (~5–10%).^63^ Together, these findings highlight scRNA-seq as a powerful tool to refine subtype classification and identify new therapeutic vulnerabilities.

**Table 2.** Molecular subtypes of pancreatic ductal adenocarcinoma and their associations with prognosis and therapeutic response.

Type	Subtypes	Markers		Prognosis	Pharmacological sensitivity	Ref
Tumor Tissue	Classical	GATA6^+^, KRAS^+^		Good	Erlotinib	[54]
	Quasimesenchymal	GATA6^‐^		Poor	Gemcitabine	
	Exocrine-like	ELA3A^+^, CFTR^+^		NR	NR	
	classical	GATA6^+^		Good	5-Fluorouracil	[55]
	basal-like	GATA6^_^		Poor	---	
	Normal	ACTA2^+^, VIM^+^, DES^+^		Good	---	
	Activated	FAP^+^		Poor	---	
	Pancreatic Progenitor	FOXA2/A3^+^, MNX1^+^, PDX1^+^, HNF4G^+^, HNF4A/1B ^+^		Good	---	[56]
	Squamous	TP63∆N^+^, MNX1^_^, NEUROD1^+^, PDX1^_^		Poor	---	
	ADEX	NR5A2^+^, MIST1^+^, RBPJL^+^, NKX2-2^+^ NEUROD1^+^		Good	---	
	Immunogenic	CD4^+^/CD8^+^ T-cell CD4^+^CD25^+^FOXP3^+^ Tregs, CTLA4^+^, PD1^+^		Good	Anti-CTLA4, Anti-PD1/PDL1	
	Pure/Immune Classic	GATA6^+^		Good	Ipilumumab	[57]
	Pure Basal-Like	CD267^+^, HACVR2^+^		Poor	ICT	
	Desmoplastic	Elastin^+^		Intermediate	FAK inhibition	
	Stroma Activated	SPARC^+^, ACTA2^+^, FAP^+^		Intermediate	FAK inhibition	
	Classical A/B	HNF1A^+^, HNF4G^+^ GATA6^+^, NKX2-2^+^ ONECUT2^+^, GATA4^+^		Good	mFOLFIRINOX	[58]
	Basal-like A/B	GATA6_		Poor	Gemcitabine	
	Hybrid	GATA6^+/_^		NR	Intermediate	
Cancer Cell	C1: LG. Ep1		Cancer-related genes↑: TFF3, EG4 Tumor suppressor genes↑: RAP1GAP cytotoxic T cells↑: CD4^+^, CD8^+^ T-cell			[60]
	C2: LG. Ep2					
	C3: LG. Ep3					
	C4: HG. Ep1		Tumor suppressor genes↓: RAP1GA Oncogenic transcripts↑: S100P, S100A10			
	C5: Myeloid cells		CD1C^+^, THBD^+^, FCER1A^+^			
	C6: Lymphocytes					
	C7: Adenocarcinoma		Tumor suppressor genes↓			
	C8: Adenocarcinoma2					
	C9: Myfibroblast1		alpha-SMA↑CXCL12 and DES↓			
	C10: Myfibeoblast2					
	C1: Type 1 ductal		KRT19^+^, CEACAM1/5/6^+^			[24]
	C2: Type 2 ductal		KRT19^++^, CEACAM1/5/6^++^			
	C3: acinar		PRSS1^+^, CELA3A^+^			
	C4: endocrine		CHGB^+^, CHGA^+^, INS^+^, IAPP^+^			
	C5: endothelial		CDH5^+^, PLVAP^+^, VWF^+^, CLDN5^+^			
	C6: fibroblast		UM^+^, DCN^+^, COL1A1^+^			
	C7: stellate		RGS5^+^, ACTA2^+^, PDGFRB^+^, ADIRF^+^			
	C8: macrophage		AIF1^+^, CD64^+^, CD14^+^, CD68^+^			
	C9: T cells		CD3D^+^, CD3E^+^, CD4^+^, CD8^+^			
	C10: B cells		MS4A1^+^, CD79A^+^, CD79B^+^, CD52^+^			
	C0		Low expressed other clusters gene markers			[63]
	C1		High expression: PDE3A, HFM1, DLG2, SLCO5A1 low expression: INO80, CSMD1			
	C2		NEAT1^+^			
	C3		ANKRD36/36C/36B^+^			
	C1: treatment enriched		Enrichment CD8^+^ T cells			[64]
	C2: squamoid-basaloid		higher epithelial and immune content lower CAF content			
	C3: classical		higher CAF and lower immune proportions			

NR: not reported.

Spatial approaches further enrich single-cell data by providing positional context. Hwang et al.^64^ combined single-nucleus RNA-seq with spatial transcriptomics in 43 PDACs, revealing three multicellular communities (treatment-enriched, squamoid-basaloid, and classical) and linking malignant programs with immune composition.^64^ This integration underscores the importance of combining scRNA-seq and ST data to capture both transcriptional diversity and tissue architecture. Collectively, these studies demonstrate that PDAC comprises diverse malignant and stromal subpopulations with distinct spatial distributions. The scRNA-seq and ST analyzes provide a mechanistic basis for how tumor heterogeneity can be translated into therapeutic strategies. Lineage plasticity between epithelial subtypes has been shown to be dynamically regulated under therapeutic pressure, where classical and quasimesenchymal states can be interconverted, contributing to treatment resistance.^65^ Stromal heterogeneity, particularly among CAF subtypes, plays a critical role in shaping the therapeutic response. Depletion of myofibroblastic CAFs has also been reported to result in more aggressive tumor phenotypes, enhanced immune evasion, and increased resistance to chemotherapy.^66^ Inflammatory CAFs promote tumor progression by establishing an immunosuppressive microenvironment, and targeting IL-6 signaling in this subset has been shown to increase the efficacy of immune checkpoint blockade.^67^ Thus, the integration of scRNA-seq with ST enables a more comprehensive understanding of tumor heterogeneity and spatial organization within the tumor microenvironment, informing precision oncology strategies and revealing novel therapeutic opportunities.

### 3.2. The tumor microenvironment in PDAC

**Figure 2.**
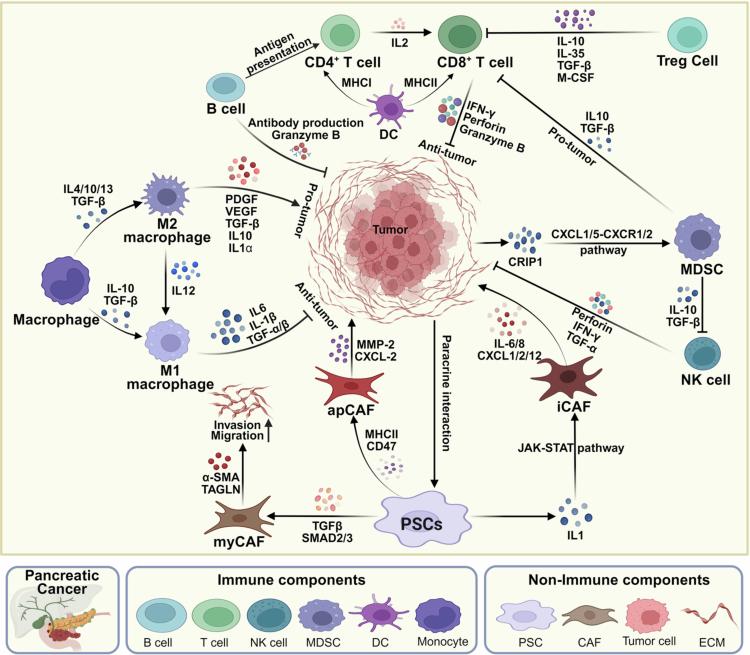
Schematic of the main components and molecular interactions in the PDAC microenvironment. The TME of PDAC comprises various cell types surrounded by a dense extracellular matrix (ECM). Activated pancreatic stellate cells (PSCs) can differentiate into distinct functional cancer-associated fibroblast (CAF) subtypes, including myofibroblast CAFs (myCAFs), inflammatory CAFs (iCAFs), and other CAFs, which collectively form a rigid barrier that supports tumor-promoting conditions and induces cancer cell proliferation. The immune cell components included B cells, T cells, natural killer (NK) cells, myeloid-derived suppressor cells (MDSCs), dendritic cells (DCs), and macrophages. Dendritic cells (DCs) initiate antitumor immunity by presenting tumor antigens to CD4⁺ T cells via MHC class II and to CD8⁺ T cells via cross-presentation through MHC class I. CD4⁺ T cells provide helper signals (e.g., IL-2) to promote CD8⁺ T-cell activation. CD8⁺ T cells exert cytotoxic effects through perforin and granzyme B expression, as well as IFN-*γ* production. B cells contribute to antibody production and antigen presentation, whereas Tregs suppress immunity through IL-10, IL-35, and TGF-*β*. M1 macrophages promote antitumor responses, whereas M2 macrophages and MDSCs support immunosuppression and tumor progression. NK cells mediate perforin-dependent cytotoxicity but are inhibited within the immunosuppressive microenvironment. Created at https://BioRender.com.

The tumor microenvironment in PDAC comprises malignant cells, stromal components, extracellular matrix (ECM), and diverse immune infiltrates, all of which interact to drive tumor progression and immune evasion.^68-70^ Importantly, this complex microenvironment represents a major source of the cellular and functional heterogeneity described above (Figure 2), where distinct tumor, stromal, and immune cell populations form spatially organized niches that influence disease progression and therapeutic response. Clinical outcomes and immunotherapy responses are strongly influenced by the composition and spatial organization of these elements. Integrating scRNA-seq with ST enables the precise dissection of cellular composition, spatial organization, and intercellular interactions within the TME, thereby revealing signaling pathways and spatially organized niches that may serve as therapeutic targets. For example, Kinker et al. demonstrated that PDAC patients with longer survival times exhibited mature tertiary lymphoid structure (TLS) signatures, with T cells producing the chemokine C-X-C motif ligand (CXCL) 13 as key organizers.^71^ Similarly, Longo et al. reported the spatial colocalization of inflammatory fibroblasts with stress-responsive cancer cells, suggesting the presence of functional stromal–epithelial crosstalk.^49^ This spatial association implies that inflammatory fibroblasts may support cancer cell adaptation to stress conditions, such as hypoxia or therapeutic pressure, thereby contributing to tumor progression and treatment resistance. Recent spatial transcriptomic studies have expanded our understanding of the spatial architecture of the PDAC tumor microenvironment and revealed distinct cellular niches that were not resolved by single-cell transcriptomics alone. Integrated single-cell and spatial transcriptomic analyzes of PDAC tissues revealed spatially organized neural invasion niches, where tertiary lymphoid structures preferentially localize near noninvaded nerves, whereas NLRP3⁺ macrophages and cancer-associated myofibroblasts accumulate around invaded nerves,^72^ highlighting the complex spatial interactions among tumor cells, immune cells and neural components during PDAC progression.

#### 3.2.1. Cancer-associated fibroblasts (CAFs)

CAFs represent the dominant nonmalignant cell type in PDAC, driving desmoplasia and immune suppression.^73^ ^,^ ^74^ Early bulk transcriptome studies first suggested the presence of stromal heterogeneity in PDAC. Subsequent scRNA-seq analyzes enabled the identification of distinct CAF subpopulations, including myofibroblastic CAFs (myCAFs), inflammatory CAFs (iCAFs), and antigen-presenting CAFs (apCAFs).^75^ ST has further refined this classification by revealing their spatial organization and interactions with tumor and immune cells within the tumor microenvironment.^76^ The functional subtypes include myCAFs, which are enriched for *α*-SMA and ECM genes and promote fibrosis. iCAFs contribute to tumor progression by inhibiting the function of cytotoxic T lymphocytes (CTLs) through the release of immunosuppressive cytokines such as IL-6, IL-10, and TGF-ß. apCAFs express MHC-II molecules with putative immunomodulatory effects.^77-79^ Targeting CAF–tumor interactions has yielded complex and sometimes paradoxical outcomes. For instance, inhibition of the Hedgehog pathway reduces the proportion of myCAFs but paradoxically enhances immunosuppressive cell infiltration, partly through upregulation of CXCL5 expression, highlighting the context-dependent role of CAFs in the tumor microenvironment.^80^ Recent spatial and multimodal transcriptomic studies have further demonstrated that stromal and immune cell populations are spatially coordinated within the PDAC microenvironment. For example, integrative scRNA-seq and high-resolution xenium spatial transcriptomics have revealed that specific fibroblast populations spatially colocalize with tumor-associated macrophages to form tumor-promoting stromal niches characterized by hypoxia, angiogenesis, and epithelial‒mesenchymal transition programs.^81^ These spatially organized interactions between stroma and immune cells contribute to tumor progression and represent potential therapeutic targets.

#### 3.2.2. Immune cells

The PDAC TME is profoundly immunosuppressive, with dysfunctional lymphocytes and suppressive myeloid populations.^7^ scRNA-seq analyzes revealed multiple subclusters, including effector, memory, Treg, and exhausted T cells.^82^ Infiltrating CD8⁺ T cells are largely dysfunctional, while Tregs accumulate, limiting antitumor responses.^83^ Regulatory pathways such as DUSP4 and IL-2R signaling further influence prognosis and therapy resistance.^84^ Therefore, T cells are considered a key mechanism in cellular immunotherapies. Interestingly, B cells are absent in early-stage PDAC but infiltrate in later stages, diversifying into subtypes with both pro- and antitumor effects.^85^ Natural killer (NK) cells and neutrophils contribute to the PDAC immune landscape. NK cells, typically defined as CD56⁺CD3^-^ innate lymphocytes, exert antitumor effects through cytotoxic activity and cytokine secretion (IFN-*γ* and TNF-*α*) and are associated with prognosis in PDAC.^86^ NK cells are frequently functionally impaired within the tumor microenvironment because of the activity of tumor- and stroma-derived factors such as TGF-*β*, leading to reduced cytotoxicity and receptor expression.^87-89^ Neutrophils are also significantly enriched in PDAC and are correlated with poor prognosis, largely because of their immunosuppressive functions, including inhibition of T-cell activity and promotion of tumor progression.^90-92^ In addition, neutrophil extracellular traps (NETs) can further impair antitumor immunity by restricting CD8⁺ T-cell and NK cell cytotoxicity.^93^ ^,^ ^94^ Myeloid cells, including macrophages, myeloid-derived suppressor cells (MDSCs), and dendritic cells (DCs), are critical components of the suppressive immune environment in the PDAC TME. DCs constitute functionally distinct subsets with context-dependent roles. Conventional DCs (cDC1s) can prime antitumor immunity. Other subsets, including tolerogenic or dysfunctional DCs, contribute to immunosuppression in the PDAC microenvironment.^95^ However, macrophages often polarize into protumor phenotypes. Beyond the classical activated macrophage (M1 macrophage)/alternatively activated macrophage (M2 macrophage) dichotomy,^96^ single-cell studies have identified additional subgroups, such as TAMs, CD169⁺, and TCR⁺ macrophages.^83^ Macrophage differentiation is influenced by tumor-derived sialic acids via Siglec receptors, promoting immunosuppression.^95^ MDSCs, expanded through CXCL1/5-CXCR1/2 signaling,^97^ ^,^ ^98^ inhibit CD4⁺/CD8⁺ T-cell and NK cell activity, further dampening antitumor immunity.^99^ ^,^ ^100^

The PDAC TME is a highly coordinated ecosystem in which CAFs remodel the stroma and shape immune exclusion, while lymphoid and myeloid cells exhibit profound dysfunction. Single-cell and spatial transcriptomic studies highlight the cellular diversity and spatial niches that underlie immunosuppression. Dissecting these interactions provides a roadmap for developing combination strategies that target stromal barriers, reprogram immune cell states, and improve the efficacy of immunotherapies. Recent spatial transcriptomic studies have also highlighted the importance of metabolic heterogeneity within the PDAC microenvironment. Spatial analysis of lactate metabolism programs revealed malignant cell subsets with hyperactive lactate metabolic pathways that promote immune evasion and tumor progression.^101^ These metabolically active tumor regions are associated with poor prognosis and may represent potential metabolic vulnerabilities for therapeutic intervention.

### 3.3. Resistance and therapeutic response in PDAC revealed by scRNA-seq and ST

Standard therapies for PDAC, such as FOLFIRINOX or gemcitabine plus nab-paclitaxel, provide limited benefit, as most patients rapidly develop resistance within six months.^102^ ^,^ ^103^ The molecular basis of this resistance is complex and multifactorial, and single-cell technologies are beginning to elucidate its mechanisms and therapeutic implications. Some scRNA-seq and ST studies of resistance and therapeutic response in PDAC are summarized in Table 3. Herein, single-cell transcriptomics has revealed key regulators of drug resistance. For instance, one-cut domain family member 2 (ONECUT2) regulates downstream KRAS signaling and is associated with poor prognosis,^104^ whereas extracellular vesicle-encapsulated miRNA-ONECUT2 promotes chemoresistance across cancers. Moreover, therapeutic resistance is frequently associated with epithelial‒mesenchymal transition (EMT)-driven tumor plasticity, which facilitates molecular subtype switching.^105^ Using scRNA-seq and ST, Kim et al. identified an EMT-associated cluster (Ep_VGLL1) that mediates the transition from classical to basal-like PDAC, suggesting a novel target for reversing resistance.^106^ Additionally, single-cell studies have highlighted TAM subsets, particularly proliferating resident macrophages, as predictors of poor response and outcome.^107^ ST approaches have not only provided important insights into therapy-induced remodeling of the PDAC microenvironment but also revealed the activation of apoptosis, the DNA damage response, and AGC-kinase signaling pathways in tumor cells after therapy. For instance, multimodal spatial analyzes comparing treatment-naïve and postneoadjuvant therapy tumors demonstrated that neoadjuvant therapy substantially remodels the tumor microenvironment by increasing the abundance of CAFs, increasing CD8⁺ T-cell infiltration, and redistributing tertiary lymphoid structures.^108^ Moreover, chemotherapy-resistant PDAC is enriched in inflammatory CAFs and transitional tumor cells with activated EMT and KRAS signaling, further underscoring the role of stromal remodeling in resistance.^109^

**Table 3.** Key single-cell and spatial transcriptomic studies revealing therapeutic resistance mechanisms and targets in PDAC.

Technique	Model system	Key findings (mechanism)	Therapeutic implications	Ref.
scRNA-seq	Mouse + Human	ONECUT2-associated acinar metaplastic programs drive tumor initiation and immune remodeling	Targeting ONECUT2-related pathways may suppress tumor progression and resistance	[104]
scRNA-seq + ST	Human	EMT-associated Ep_VGLL1 tumor cell cluster mediates subtype transition and chemoresistance	Targeting EMT-driven plasticity may reverse resistance	[106]
scRNA-seq + multiomics	Human + Mouse	Proliferating resident macrophages promote chemoresistance via metabolic adaptation and immunosuppression	Targeting TAM subsets restores chemotherapy sensitivity and reduces fibrosis	[107]
snRNA-seq + ST	Human	Neural-like progenitor malignant cell program enriched after therapy and associated with poor prognosis.	Identifies therapy-resistant malignant states for precision targeting	[64]
scRNA-seq + ST	Human	Spatial heterogeneity of CAFs (tumor-proximal vs distal) regulates immune response and clinical outcome	CAF-targeted strategies may improve prognosis and therapeutic response	[110]
scRNA-seq + snRNA-seq + ST	Human	Chemotherapy-resistant PDAC exhibits enrichment of inflammatory CAFs and transitional tumor cell states with EMT and KRAS signaling activation; associated with immune evasion	Targeting CAF-mediated stromal remodeling and immune checkpoint pathways may overcome resistance	[109]

Despite the central role of programmed death 1 (PD-1) and cytotoxic T lymphocyte-associated protein 4 (CTLA-4) blockade in cancer, PDAC remains refractory to immune checkpoint inhibitors.^111^ This failure is driven by its fibrotic stroma, dense MDSC infiltration, and immunosuppressive TME, and the classification of PDAC as a “cold tumor”.^112^ However, scRNA-seq has illuminated strategies to remodel the TME and restore responsiveness. For example, targeting CAF heterogeneity can increase immunotherapy efficacy, as CAF modulation promotes vascular remodeling and increases CD8⁺ T-cell infiltration.^113^ Constitutive activation of KRAS, a hallmark of PDAC, not only drives tumor cell proliferation through MAPK signaling but also promotes metabolic reprogramming and suppresses MHC class I expression, thereby contributing to immune evasion and resistance to therapy.^114^ Recent advances in targeting oncogenic KRAS have opened new avenues for PDAC treatment. Although allele-specific inhibitors targeting KRAS G12C (sotorasib and adagrasib) have demonstrated clinical efficacy in other solid tumors, their applicability in PDAC is limited because of the low prevalence of KRAS G12C mutations.^115^ Pan-KRAS inhibitors such as BI-2865 and multiple RAS (ON) inhibitors (RMC-6236) have demonstrated promising preclinical and early clinical activity across KRAS-mutant tumors, indicating that broad-spectrum RAS inhibition suppressed ERK signaling and induced apoptosis in PDAC.^116^ ^,^ ^117^ Although previous attempts to target KRAS signaling have often been limited by compensatory pathway activation and toxicity, underscoring the need for combinatorial therapeutic strategies, single-cell and spatial transcriptomic analyzes may provide critical insights into KRAS-driven tumor heterogeneity, stromal interactions, and immune evasion, thereby offering a rationale for combining KRAS-targeted therapies with immunotherapy or stromal-targeting approaches to overcome resistance in PDAC.

Integration of scRNA-seq and ST provides high-resolution maps of ligand–receptor networks that mediate resistance.^68^ Hwang et al. reported that a neural-like progenitor malignant cell program enriched after chemotherapy was linked to poor prognosis.^64^ Specific interactions, such as ligand galectin-9 (LGALS9)-CD44, which drives CD8⁺ T-cell exhaustion and MDSC expansion,^118^ ^,^ ^119^ and osteopontin (SPP1)-mediated immune evasion,^120^ highlight novel checkpoint pathways beyond PD-1/CTLA-4. Moreover, spatial transcriptomic analyzes revealed that inflammatory CAF subsets localized in tumor-distal regions exhibit complement-associated gene signatures, linking stromal heterogeneity to both prognosis and therapeutic resistance.^109^ ^,^ ^110^ These findings underscore CAFs as key regulators of treatment response and support combination strategies targeting both stromal and immune compartments. Spatial transcriptomics has also enabled the identification of predictive biomarkers. High infiltration of T cells and monocytes is correlated with an improved response to neoadjuvant therapy and prolonged survival, highlighting spatial immune features as predictive biomarkers.^121^ In summary, single-cell and spatial transcriptomic studies have demonstrated that PDAC resistance arises from tumor cell plasticity, immunosuppressive macrophage subsets, and stromal barriers that limit effective antitumor immunity. These technologies further provide a framework for rational combination therapies, including CAF reprogramming, metabolic targeting, and novel immune checkpoint inhibition, to enhance therapeutic response.

## 4. Conclusion and future directions

Although scRNA-seq and ST provide unbiased, transcriptome-wide characterization of cellular states and intercellular communication networks, they lack direct morphological context. Emerging digital pathology approaches provide a complementary framework to sequencing-based technologies for characterizing PDAC. Conventional histopathology, particularly hematoxylin and eosin (H&E) staining, remains the clinical gold standard for tumor diagnosis and tissue architecture assessment, whereas recent advances in high-plex imaging and label-free techniques have enabled the extraction of quantitative, spatially resolved morphological features, which preserved intact tissue architecture and was compatible with formalin-fixed paraffin-embedded (FFPE) specimens, thereby enabling large-scale clinical applicability and retrospective analyzes.^122^ ^,^ ^123^ Multiplex immunofluorescence and whole-slide imaging by integrating imaging mass spectrometry, immunofluorescence, and histology can generate high-dimensional spatial maps of immune infiltration and tumor cell states and reveal the heterogeneous intratumoral distribution of chemotherapeutic agents.^124^ ^,^ ^125^ Together, these findings underscore that imaging-based pathology excels in resolving tissue architecture and spatial drug distribution, whereas sequencing approaches define molecular programs and cellular heterogeneity. Therefore, integrating scRNA-seq and ST with digital pathology using AI technology in the future represents a powerful strategy to bridge morphology and molecular biology, enabling more accurate mapping of tumor architecture, refinement of cellular heterogeneity, and improved prediction of therapeutic response, which may further refine and potentially redefine transcriptomic subtypes of PDAC.

Building upon these advances, the introduction of single-cell RNA sequencing and spatial transcriptomics has revolutionized our ability to analyze the cellular and molecular intricacies of PDAC. By combining transcriptional profiles with spatial information, these advanced methodologies offer unparalleled resolution in understanding tumor heterogeneity, tumor-stromal interactions, and the immunosuppressive mechanisms that contribute to therapeutic resistance. These insights enhance the classification of PDAC subtypes, refine prognostic predictions, and identify novel cellular targets for therapeutic intervention. Although genomic approaches define the static mutational architecture of PDAC, such as KRAS-driven oncogenic programs and proteomic analyzes, which capture functional protein abundance and posttranslational modifications, transcriptomic profiling occupies a central position within the broader multiomics landscape and provides a dynamic and cell type-resolved view of gene expression programs, bridging the gap between genotype and phenotype. Thus, the scRNA-seq and ST approaches are uniquely capable of resolving cellular heterogeneity, transient cell states, and context-dependent signaling interactions in PDAC that cannot be captured by bulk genomics or proteomics alone. In the future, several pivotal directions are anticipated to expedite advancements in the field. First, the integration of single-cell RNA sequencing and spatial transcriptomics with multiomics such as epigenomics, proteomics, and metabolomics, is expected to provide a comprehensive systems-level understanding of pancreatic ductal adenocarcinoma biology. Such integrative strategies using AI technology will enable the mapping of regulatory cascades from genetic alterations to transcriptional programs and downstream functional networks, thereby improving mechanistic interpretability. Second, longitudinal and treatment-responsive single-cell studies are essential for elucidating tumor evolution, therapy-induced plasticity, and resistance mechanisms in real time. Third, the translation of these findings into clinical applications will necessitate functional validation and the use of patient-derived models to evaluate the therapeutic potential of candidate pathways, such as epithelial‒mesenchymal transition regulators, cancer-associated fibroblast subtypes, and novel immune checkpoints. Finally, as computational methodologies advance, artificial intelligence-driven multimodal data integration may facilitate the development of predictive biomarkers and inform personalized therapeutic strategies.

In conclusion, the integration of single-cell and spatial transcriptomics significantly enhances our mechanistic understanding of pancreatic ductal adenocarcinoma and has the potential to revolutionize its management. As the central layer links genomic alterations to proteomic and phenotypic outcomes, transcriptomics provides a critical framework for translating molecular insights into clinically actionable strategies. This advancement may lead to improved early detection and the development of rational combination therapies, ultimately facilitating more precise and effective interventions against this challenging disease.

## Data Availability

No data were used for the research described in the article.

## Abbreviations

ADEX: aberrantly differentiated endocrine exocrine
ApCAFs: antigen-presenting CAFs
Bulk RNA-seq: bulk RNA transcriptome sequencing
CAFs: cancer-associated fibroblasts
cDC1s: conventional DCs
CEL-Seq: cell expression by linear amplification and sequencing
CTLA-4: cytotoxic T lymphocyte-associated protein 4
CXCL: chemokine C-X-C motif ligand
DCs: dendritic cells
Drop-seq: Droplet sequencing
ECM: extracellular matrix
EMT: epithelial–mesenchymal transition
ESCC: esophageal squamous cell carcinoma
iCAFs: inflammatory CAFs
InDrop: indexing droplets
IPMN: intraductal papillary mucinous neoplasm
MARS-seq: massively parallel scRNA-seq
ISH: in situ hybridization
ISS: in situ sequencing
LCM: laser capture microdissection
LGALS9: ligand galectin-9
MDSCs: myeloid-derived suppressor cells
MERFISH: multiplexed error-robust fluorescence ISH
MIA: multimodal intersection analysis
myCAFs: myofibroblast CAFs
NETs: neutrophil extracellular traps
NGS: next-generation sequencing
NK: natural killer
NMF: nonnegative matrix factorization
ONECUT2: one cut domain family member 2
PaCa: pancreatic cancer
PD-1: programmed death 1
PDAC: pancreatic ductal adenocarcinoma
SCRB-seq: single-cell RNA barcoding and sequencing
scRNA-seq: single-cell RNA sequencing
Smart-seq: switching mechanism at the 5’ end of the RNA transcript
ST: spatial transcriptomics
STARmap: spatially resolved transcript amplicon readout mapping
STRT-seq: single-cell tagged reverse transcription sequencing
TME: tumor microenvironment
